# Effects of salicylic acid, zinc and glycine betaine on morpho-physiological growth and yield of maize under drought stress

**DOI:** 10.1038/s41598-021-82264-7

**Published:** 2021-02-04

**Authors:** Ramadan Shemi, Rui Wang, El-Sayed M. S. Gheith, Hafiz Athar Hussain, Saddam Hussain, Muhammad Irfan, Linna Cholidah, Kangping Zhang, Sai Zhang, Longchang Wang

**Affiliations:** 1 College of Agronomy and Biotechnology, Southwest University, Chongqing, 400715 China; 2 Department of Agronomy, Faculty of Agriculture, Cairo University, Giza, 12613 Egypt; 3 Institute of Environment and Sustainable Development in Agriculture, Chinese Academy of Agricultural Sciences, Beijing, 100081 China; 4 Department of Agronomy, University of Agriculture, Faisalabad, 38040 Pakistan; 5 Department of Agronomy, Zakaria University, Multan, 60800 Pakistan

**Keywords:** Plant sciences, Plant stress responses, Drought

## Abstract

Drought is one of the major environmental stresses that negatively affect the maize (*Zea mays* L.) growth and production throughout the world. Foliar applications of plant growth regulators, micronutrients or osmoprotectants for stimulating drought-tolerance in plants have been intensively reported. A controlled pot experiment was conducted to study the relative efficacy of salicylic acid (SA), zinc (Zn), and glycine betaine (GB) foliar applications on morphology, chlorophyll contents, relative water content (RWC), gas-exchange attributes, activities of antioxidant enzymes, accumulations of reactive oxygen species (ROS) and osmolytes, and yield attributes of maize plants exposed to two soil water conditions (85% field capacity: well-watered, 50% field capacity: drought stress) during critical growth stages. Drought stress significantly reduced the morphological parameters, yield and its components, RWC, chlorophyll contents, and gas-exchange parameters except for intercellular CO_2_ concentration, compared with well water conditions. However, the foliar applications considerably enhanced all the above parameters under drought. Drought stress significantly (p < 0.05) increased the hydrogen peroxide and superoxide anion contents, and enhanced the lipid peroxidation rate measured in terms of malonaldehyde (MDA) content. However, ROS and MDA contents were substantially decreased by foliar applications under drought stress. Antioxidant enzymes activity, proline content, and the soluble sugar were increased by foliar treatments under both well-watered and drought-stressed conditions. Overall, the application of GB was the most effective among all compounds to enhance the drought tolerance in maize through reduced levels of ROS, increased activities of antioxidant enzymes and higher accumulation of osmolytes contents.

## Introduction

Maize (*Zea mays* L.) is one of the most important cereal crops globally. Maize is as the major source of food, feed, and bio-fuel and therefore, consumption and demand of maize is increasing worldwide^1^. In China, maize is the third-most important food crop after rice and wheat. The cultivated area for maize in China is estimated at 42.42 million ha with yield of about 259.23 million tonnes year^1,2^. However, it is an extremely sensitive cereal crop to drought stress, especially during critical growth stages. It has been well reported that maize is comparatively more vulnerable to drought stress compared with other cereals such as wheat^3^.

Globally, the demands for food crops are projected to be doubled by 2050 because of the ever increasing and burgeoning population^4^. The irrigation water is considered a major resource to crop production, but it is scarce and expensive in many regions of the world, as a result, maize production is often restricted. Universally, the agriculture sector utilizes around 70% of freshwater for irrigation to the farming lands^5^. Approximately 40% of the total food is produced from about 17% of the cropped land area by irrigated agriculture^6^. With the continued increase of world population, industrialization, and urbanization, the competition for fresh water will be highly increased among agriculture and other sectors^4,7^.

Drought stress severely hampers the growth and productivity of maize^8–10^. It triggers different changes in crop plants through various morphological, physiological, and biochemical responses^11–13^. Drought stress causes oxidative damage in plants through higher production of ROS, nevertheless, plants possess the antioxidant defense system and enhanced synthesis of antioxidants such as the ascorbate peroxidase (APX), glutathione reductase (GR), peroxidase (POD), superoxide dismutase (SOD), and catalase (CAT) helps in quenching of ROS produced during drought stress ^10,13–17^.The exogenous applications of plant growth regulators, micronutrients, or osmoprotectants can play a significant role in improving drought-resistance at various plant growth stages^18,19^. Salicylic acid (SA) is a natural phenolic compound that can effectively alleviate the harmful effects caused by abiotic environmental stresses^20,21^. Moreover, it can play important role in modulating growth, and physiological and biochemical characteristics in plants^17,22–24^. The SA has been reported to be beneficial in relieving the negative effects of drought stress by improving the seedling growth, leaf gas-exchange traits, and activities of APX, CAT and SOD enzymes, while decreasing the MDA and H_2_O_2_ contents^25,26^. Sharma et al.^27^ suggested that SA markedly increased drought-tolerance and could be used for increasing and stabilizing crop production under stress conditions. Zinc (Zn) is an essential micronutrient that is involved in physiological functions and structure of the regulatory cofactor of many enzymes, carbohydrate and chlorophyll production, pollen development, fertilization, RNA and DNA metabolism, and protein synthesis^28,29^. Zn plays a critical role in improving resistance against drought stress by detoxifying ROS generation and increasing antioxidant enzymes^30,31^. Glycine betaine (GB) is naturally occurring osmoprotectant compound that accumulates in numerous plants under drought stress. Iqbal et al.^32^ reported that foliar application of GB improved turgor potential and sunflower yield under drought stress. Raza et al.^33^ indicated that the growth parameters and yield components were reduced under drought stress in wheat plants, while exogenous application of GB was effective in mitigating the detrimental effects of drought. Hasanuzzaman et al.^34^ recorded that the GB played an important role in improving the detoxification of ROS, hence recovering photosynthesis and decreasing oxidative damage.

Nevertheless, no study has been conducted to appraise the relative effect of these compounds (SA, Zn, or GB) in improving maize tolerance against drought stress. The present study was conducted to assess the possible role of SA, Zn, anGB betaine treatments in improving drought-tolerance in maize under critical growth stages, based on changes in growth, yield, and physiological and biochemical features. It was hypothesized that the exogenous applications of these compounds could mitigate the negative effects of drought stress in maize from the fourteenth leaf (V_14_) until blister (R_2_) growth stages by regulating antioxidant enzymes. To verify this hypothesis, the maize plants were sprayed with SA, Zn, and GB under well-watered and drought-stressed conditions. The specific objectives were to (1) examine the response of maize growth characters, yield, and its components to the foliar application of SA, Zn, and GB under different soil water conditions during critical growth stages; (2) investigate the effect of respective spraying applications on chlorophyll contents, RWC, leaf gas-exchange attributes, ROS accumulation, MDA content, activity of antioxidant enzymes, and osmolytes accumulation under different soil water conditions; and (3) compare the relative efficacy of different spraying applications for ameliorating the harmful effects of drought stress by enhancing the preceding parameters in maize.

## Results

### Growth parameters, yield, and its components

Drought stress significantly (p < 0.05) disrupted the maize growth parameters, yield, and its components in terms of plant height, fresh weight plant^−1^, dry weight plant^−1^, the number of leaves plant^−1^, leaf area plant^−1^, the number of grains plant^−1^, 100-grain weight, biological yield plant^−1^, grain yield plant^−1^, and harvest index. However, the results showed that the growth parameters, yield, and its components were improved by SA, Zn, and GB spraying treatments under well-watered and drought-stressed conditions (Tables 1, 2, 3). As predicted, one or more of spraying treatments statistically improved all the above parameters in both soil water conditions except for plant height and harvest index under the well-watered condition as compared with the control treatment. Under the drought-stressed condition, the SA, Zn, or GB spray increased the plant height by 7.32%, 5.50%, and 10.07%, fresh weight by 23.08%, 16.82%, and 25.34%, dry weight by 14.31%, 12.28%, and 22.55%, number of leaves by 14.35%, 8.65%, and 25.56%, leaf area by 17.22%, 11.54%, and 22.81%, number of grains by 16.03%, 2.48%, and 29.51%, 100-grain weight by 20.83%, 16.30%, and 23.46%, biological yield by 27.54%, 12.48%, and 41.10%, grain yield by 40.52%, 19.98%, and 60.49%, and harvest index by 10.12%, 6.46%, and 13.65%, respectively, as compared to the values of control. Generally, the maximum growth parameters, yield, and its components were recorded from the plants treated with GB followed by SA and Zn spraying treatments under two soil water conditions.

**Table 1:** Effect of soil water conditions and foliar treatments on plant height, fresh weight, dry weight, number of leaves, and leaf area.

Soil water conditions	Foliar treatments	Plant height (cm)	Fresh weight plant^−1^ (g)	Dry weight plant^−1^ (g)	No. of leaves plant^−1^	Leaf area plant^−1^ (cm^2^)
WW	CK	181.00^ab^ ± 5.69	337.87^b^ ± 19.49	63.71^bc^ ± 2.83	12.89^bc^ ± 0.49	5237.47^bc^ ± 125.44
	SA	186.67^a^ ± 5.24	362.89^a^ ± 16.10	68.16^ab^ ± 2.16	14.44^ab^ ± 0.29	5775.23^a^ ± 145.49
	Zn	184.33^a^ ± 6.37	350.74^ab^ ± 11.55	66.91^ab^ ± 2.09	14.33^ab^ ± 0.33	5605.47^ab^ ± 147.14
	GB	187.67^a^ ± 3.72	368.65^a^ ± 22.74	70.63^a^ ± 2.53	14.85^a^ ± 0.46	5969.70^a^ ± 234.39
	Means	184.91	355.03	67.35	14.13	5646.96
WD	CK	109.32^d^ ± 4.49	191.64^e^ ± 8.89	37.86^ fg^ ± 1.21	9.82^e^ ± 0.61	3703.93f. ± 180.88
	SA	117.33^c^ ± 3.29	235.88^c^ ± 11.73	43.28^de^ ± 2.14	11.23^ cd^ ± 0.62	4341.98^de^ ± 229.12
	Zn	115.34^ cd^ ± 3.93	223.88^ cd^ ± 10.90	42.51^ef^ ± 2.41	10.67^de^ ± 0.33	4131.53^e^ ± 271.97
	GB	120.33^c^ ± 3.18	240.21^c^ ± 9.90	46.40^d^ ± 2.53	12.33^c^ ± 0.67	4548.87^d^ ± 165.13
	Means	115.58	222.90	42.51	11.01	4181.57

Values are means (± SE) of three replicates. For L.S.D.’s results, means with different letters indicate that means are different at 95% confidence level.

WW, well-watered; WD, water-deficient; CK, control (double distilled water); SA, salicylic acid; Zn, Zinc; GB, Glycine betaine.

**Table 2:** Effect of soil water conditions and foliar treatments on the number of grains, 100-grain weight, biological yield, grain yield, and harvest index.

Soil water conditions	Foliar treatments	No. of grains plant^−1^	100-grain weight (g)	Biological yield plant^−1^ (g)	Grain yield plant^−1^ (g)	Harvest index (%)
WW	CK	294.65^bc^ ± 15.25	31.18^b^ ± 0.98	256.35^c^ ± 4.04	91.72^bc^ ± 3.93	35.76^a^ ± 1.20
	SA	312.10^ab^ ± 15.14	32.55^b^ ± 0.64	273.13^ab^ ± 5.20	101.40^ab^ ± 2.99	37.12^a^ ± 0.84
	Zn	302.80^b^ ± 16.30	32.22^b^ ± 1.25	265.07^bc^ ± 5.82	97.15^b^ ± 1.65	36.71^a^ ± 1.37
	GB	324.19^a^ ± 25.93	33.20^a^ ± 2.13	284.32^a^ ± 6.01	106.53^a^ ± 1.28	37.48^a^ ± 0.34
	Means	308.43	32.28	269.71	99.19	36.76
WD	CK	215.78^ef^ ± 14.79	24.72^ cd^ ± 1.04	163.33^ g^ ± 3.78	53.05f. ± 1.70	32.51^b^ ± 1.16
	SA	250.36^d^ ± 12.43	29.87^bc^ ± 1.01	208.32^e^ ± 5.12	74.55^d^ ± 1.80	35.80^a^ ± 0.51
	Zn	221.13^e^ ± 5.99	28.75^c^ ± 0.76	183.71f. ± 5.87	63.65^e^ ± 3.11	34.61^ab^ ± 0.63
	GB	279.46^c^ ± 10.02	30.52^b^ ± 0.93	230.46^d^ ± 4.71	85.14^c^ ± 2.01	36.95^a^ ± 0.50
	Means	241.68	28.46	196.45	69.09	34.96

Values are means (± SE) of three replicates. For L.S.D.’s results, means with different letters indicate that means are different.

at 95% confidence level.

WW, well-watered; WD, water-deficient; CK, control (double distilled water); SA, salicylic acid; Zn, Zinc; GB, Glycine betaine.

**Table 3:** p-values of the two-way factorial analysis of growth, yield, and physiological and biochemical parameters of maize as influenced by various foliar treatments under both soil water conditions.

Parameters	Main factors effects		Interaction effects
	S	F	S × F
Plant height	< 0.0001	< 0.0293	< 0.0491
Fresh weight	< 0.0001	< 0.0069	< 0.0426
Dry weight	< 0.0001	< 0.0281	< 0.0487
Number of leaves	< 0.0001	< 0.0060	< 0.0268
Leaf area	< 0.0001	< 0.0030	< 0.0376
Number of grains	< 0.0001	< 0.0340	< 0.0473
100-grain weight	< 0.0003	< 0.0204	< 0.0342
Biological yield	< 0.0001	< 0.0001	< 0.0073
Grain yield	< 0.0001	< 0.0001	< 0.0152
Harvest index	< 0.0113	< 0.0197	< 0.0466
Chlorophyll a content	< 0.0022	< 0.0014	< 0.0327
Chlorophyll b content	< 0.0001	< 0.0078	< 0.0254
Total chlorophyll content	< 0.0001	< 0.0001	< 0.0426
RWC	< 0.0001	< 0.0273	< 0.0305
Net photosynthesis rate	< 0.0001	< 0.0001	< 0.0254
Transpiration rate	< 0.0001	< 0.0001	< 0.0159
Stomatal conductance	< 0.0001	< 0.0001	< 0.0289
Intercellular CO_2_ concentration	< 0.0001	< 0.0001	< 0.0186
APX activity	< 0.0001	< 0.0001	< 0.0008
GR activity	< 0.0001	< 0.0001	< 0.0001
POD activity	< 0.0003	< 0.0001	< 0.0376
CAT activity	< 0.0001	< 0.0001	< 0.0226
SOD activity	< 0.0001	< 0.0039	< 0.0288
MDA content	< 0.0001	< 0.0001	< 0.0342
H_2_O_2_ content	< 0.0001	< 0.0001	< 0.0245
O_2_^−^ content	< 0.0001	< 0.0001	< 0.0076
Free proline content	< 0.0001	< 0.0001	< 0.0008
Total soluble sugar	< 0.0001	< 0.0001	< 0.0287

‘S’: effect of soil water conditions; ‘F’: effect of foliar treatments; S × F, effect of the interaction between two variables.

p-values are regarded as significant (p < 0.05, n = 3) and highly significant (p < 0.01, n = 3).

### Chlorophyll contents and RWC

**Figure 1:**
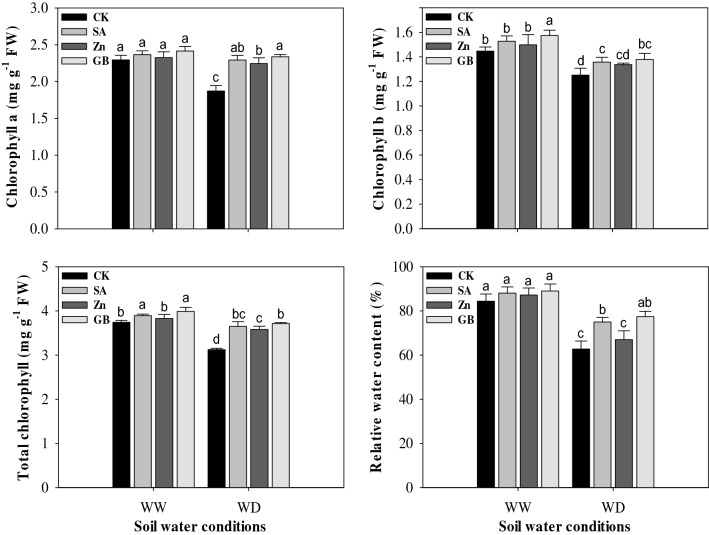
Effect of soil water conditions and foliar treatments on chlorophyll a, chlorophyll b, total chlorophyll, and relative water contents. Every column in each graph represents the mean (± SE) of three replicates. Different letters above columns indicate that means are different at 95% confidence level. WW, well-watered; WD, water-deficient; CK, control (double distilled water); SA, salicylic acid; Zn, Zinc; GB, Glycine betaine.

Drought stress statistically (p < 0.05) reduced the chlorophyll (Chl.) contents of maize leaves and relative water content (RWC). However, the results revealed that the Chl. a, Chl. b, and total Chl. contents and RWC were improved by SA, Zn, and GB spraying treatments in both soil water conditions (Fig. 1 and Table 3). Compared with control, Chl. a content was significantly increased by spraying treatments under the drought-stressed condition. Chl. b content was substantially enhanced by GB treatment under both soil water conditions and by SA treatment under the drought-stressed condition. Total Chl. content was statistically influenced by spraying treatments under both soil water conditions except for Zn treatment under the well-watered condition. RWC was statistically affected by GB and SA treatments under the drought-stressed condition. Under the drought-stressed condition, the concerned spraying treatments improved Chl. a content by 22.45%, 19.78%, and 24.59%, Chl. b content by 8.00%, 6.40%, and 10.40%, total Chl. content by 16.98%, 14.74%, and 18.91%, and RWC by 19.44%, 6.75%, and 23.42%, respectively, as compared to the values of the control treatment. Overall, maximum chlorophyll contents and RWC were registered from the plants treated with GB and followed by SA and Zn spraying treatments under both soil water conditions.

### Leaf gas-exchange attributes

**Figure 2:**
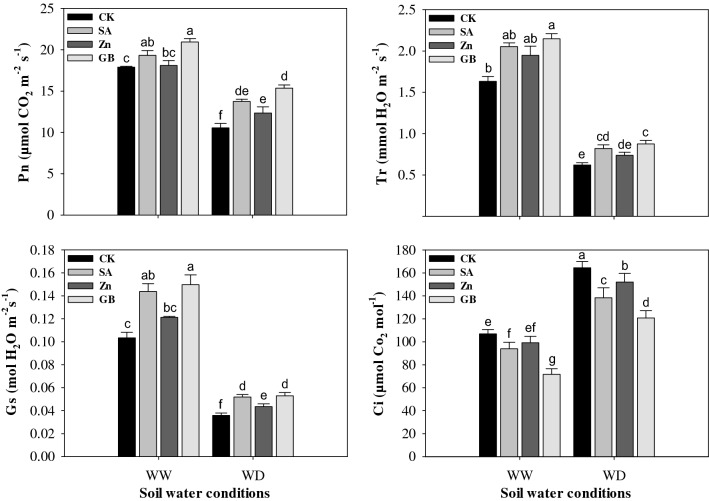
Effect of soil water conditions and foliar treatments on net photosynthesis rate (Pn), transpiration rate (Tr), stomatal conductance (Gs), and intercellular CO_2_ concentration (Ci). Every column in each graph represents the mean (± SE) of three replicates. Different letters above columns indicate that means are different at 95% confidence level. WW, well-watered; WD, water-deficient; CK, control (double distilled water); SA, salicylic acid; Zn, Zinc; GB, Glycine betaine.

Drought stress significantly (p < 0.05) influenced the net photosynthesis rate (Pn), transpiration rate (Tr), stomatal conductance (Gs), and intercellular CO_2_ concentration (Ci). However, results noticed that the spraying treatments of SA, Zn, and GB increased the above leaf gas-exchange parameters except for Ci (Fig. 2 and Table 3). Pn and Gs were significantly increased by spraying treatments under both soil water conditions except for Zn treatment under the well-watered condition. Tr was statistically enhanced by GB treatment under both soil water conditions and by SA treatment under the drought-stressed condition. Ci was statistically decreased by spraying treatments under both soil water conditions except for Zn treatment under the well-watered condition. Under the drought-stressed condition, the respective spraying treatments promoted the Pn by 30.48%, 17.09%, and 45.77%, Tr by 32.25%, 19.35%, and 40.32%, and Gs by 66.66%, 33.33%, and 66.66%; while they decreased the Ci by 15.91%, 7.59%, and 26.62%, respectively, as compared to the values of the control. Overall, the spraying treatments improved the leaf gas-exchange attributes under the drought-stressed condition more than the well-watered condition. The GB treatment was more effective than SA and Zn treatments as compared with control under both soil water conditions.

### Antioxidant enzymes activity and MDA content

**Figure 3:**
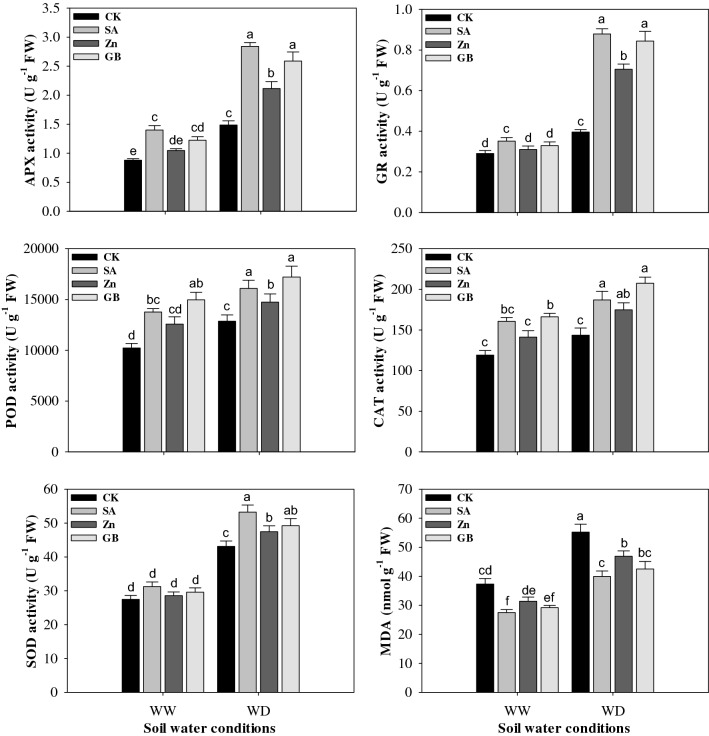
Effect of soil water conditions and foliar treatments on the activities of ascorbate peroxidase (APX), glutathione reductase (GR), peroxidase (POD), catalase (CAT) and superoxide dismutase (SOD), and malonaldehyde (MDA) content. Every column in each graph represents the mean (± SE) of three replicates. Different letters above columns indicate that means are different at 95% confidence level. WW, well-watered; WD, water-deficient; CK, control (double distilled water); SA, salicylic acid; Zn, Zinc; GB, Glycine betaine.

Drought stress significantly (p < 0.05) increased the MDA content and triggered the activities of APX, GR, POD, CAT, and SOD. However, application of SA, Zn, and GB increased the activities of APX, GR, POD, CAT, and SOD, but reduced the content of MDA under both soil water conditions (Fig. 3 and Table 3). Compared with control, the activities of APX and POD were statistically increased by spraying treatments under both soil water conditions except for Zn treatment under the well-watered condition, the GR activity was significantly enhanced by spraying treatments under the drought-stressed condition and by SA treatment under the well-watered condition, the CAT activity was statistically improved by spraying treatments under the drought-stressed condition and by GB treatment under the well-watered condition, the SOD activity was statistically increased by spraying treatments under the drought-stressed condition, but the MDA content was significantly decreased by spraying treatments under both soil water conditions except for Zn treatment under the well-watered condition. Under the drought-stressed condition, the concerned spraying treatments improved the activities of APX by 91.89%, 42.56%, and 74.32%, GR by 125.64%, 79.48%, and 115.38%, POD by 25.16%, 14.63%, and 33.91%, CAT by 30.23%, 21.80%, and 44.54%, SOD by 23.54%, 10.06%, and 14.17%, but reduced the MDA content by 27.70%, 15.13%, and 23.01%, respectively, as compared to the values of the control. Overall, the respective spraying treatments enhanced all the antioxidant enzymes activity and reduced the MDA content under the drought-stressed condition more than the well-watered condition. Results noticed that the SA treatment was the most effective and followed by GB and Zn spraying treatments in decreasing the content of MDA and increasing the activities of APX, SOD, and GR enzymes; while the GB treatment had higher values more than other spraying treatments in raising the activity of POD and CAT enzymes as compared with control treatment under both soil water conditions.

### Accumulation of reactive oxygen species

**Figure 4:**
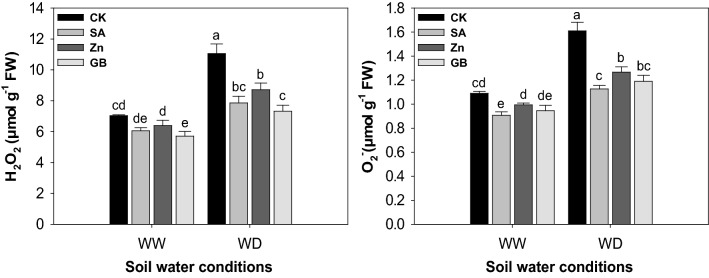
Effect of soil water conditions and foliar treatments on hydrogen peroxide (H_2_O_2_) and superoxide anion (O_2_^−^) contents. Every column in each graph represents the mean (± SE) of three replicates. Different letters above columns indicate that means are different at 95% confidence level. WW, well-watered; WD, water-deficient; CK, control (double distilled water); SA, salicylic acid; Zn, Zinc; GB, Glycine betaine.

Drought stress significantly (p < 0.05) increased the level of ROS accumulation. However, the spraying treatments of SA, Zn, and GB reduced the contents of hydrogen peroxide (H_2_O_2_) and superoxide anion (O_2_^−^) in both soil water conditions (Fig. 4 and Table 3). The H_2_O_2_ content was significantly decreased by spraying treatments under the drought-stressed condition and by GB treatment under the well-watered condition. The O_2_^−^ content was statistically declined by spraying treatments under the drought-stressed condition and by SA treatment under the well-watered condition. Under the drought-stressed condition, application of SA, Zn, and GB decreased the contents of H_2_O_2_ by 28.92%, 21.18%, and 33.81%, and O_2_^−^ by 30.43%, 21.73%, and 26.08%, respectively relative to the values of control. Overall, the positive effect of spraying treatments was better under drought-stressed than well-watered conditions.

### Accumulation of osmolytes

**Figure 5:**
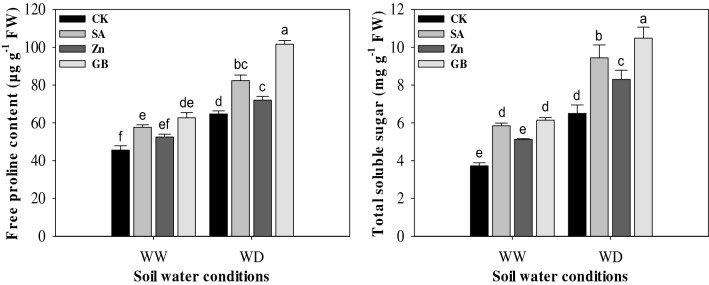
Effect of soil water conditions and foliar treatments on the accumulations of free proline content and total soluble sugar. Every column in each graph represents the mean (± SE) of three replicates. Different letters above columns indicate that means are different at 95% confidence level. WW, well-watered; WD, water-deficient; CK, control (double distilled water); SA, salicylic acid; Zn, Zinc; GB, Glycine betaine.

Drought stress significantly (p < 0.05) affected the free proline content and total soluble sugar. However, the spraying treatments of SA, Zn, and GB enhanced the proline content and soluble sugar under both soil water conditions (Fig. 5 and Table 3). Compared with control, the free proline content and total soluble sugar were significantly increased by spraying treatments under two soil water conditions except for Zn treatment under the well-watered condition. Under the drought-stressed condition, the concerned spraying treatments enhanced the free proline content by 27.32%, 11.25%, and 57.08%, and total soluble sugar by 45.23%, 27.53%, and 61.23%, respectively, as compared to the values of the control. Overall, the positive effects of spraying treatments were better under the drought-stressed condition than the well-watered condition; the GB treatment was more effective in increasing the proline content and soluble sugar than other spraying treatments as compared with control treatment under both soil water conditions.

## Discussion

Drought stress is a key agricultural threat that negatively impacts the crop production. It may lead to an imbalance between the ROS accumulation and defense systems of antioxidant, resulting in oxidative damage^17,35–38^. Drought stress may inhibit the growth and development of numerous crops, yet the reproductive growth phases are highly sensitive by drought stress conditions^38–40^. In the present study, results (Tables 1, 2, 3) indicated that drought stress significantly (p < 0.05) disrupted the maize growth parameters, yield, and yield components. However, one or more spraying treatments statistically enhanced the growth parameters, yield, and its components except for the plant height and harvest index. Previously, Anjum et al.^41^ reported that the progressive drought condition significantly diminished the plant height, number of ears, fresh and dry weight of shoot, and grain yield when compared with well-watered plants in two cultivars of maize. Ullaha et al.^42^ concluded that the application of adequate Zn treatment significantly increased the area of leaves and dry weight of seedlings of chickpea under drought stress. Furthermore, Osman^43^ reported that the growth parameters, yield, and yield-related components were considerably reduced under drought stress; while the exogenous application of GB statistically stimulated the leaves number plant^−1^, fresh leaf weight plant^−1^, pods number plant^−1^, and green pods yield of pea under drought stress when compared with control. Ghazi^44^ recorded that the drought stress statistically decreased the growth and yield parameters; while the exogenous of SA treatment increased the plant height, fresh and dry weight of flag leaf, cob length, grains number, 100-grain weight, and grain, straw and cob yields under drought stress as compared with the untreated plants with SA. In our study, the decreases in maize growth parameters, yield, and yield components under drought stress might be attributed to the overproduction of ROS (H_2_O_2_ and O_2_^−^) which caused oxidative damage to the membranes, lipids and elevated the content of MDA (Figs. 3, 4). Several previous studies have documented that drought stress leads to an increase in the ROS production that destroys the cell membrane, causes damage to lipids, proteins, and chlorophylls, and finally decreases plant biomass accumulation^15,45^. However, the present study showed that the exogenous applications of SA, Zn, and GB could improve drought-tolerance in maize and the improvement might be attributed to increase in Chl. a, Chl. b, and total Chl., and RWC (Fig. 1), enhanced leaf gas-exchange attributes (Fig. 2), improved antioxidant enzymes activities (Fig. 3), reduced MDA, H_2_O_2_, and O_2_^−^ contents (Figs. 3, 4), and increased accumulation of osmolytes (Fig. 5), particularly under the drought-stressed condition. Growth improvement and yield enhancement by these spraying applications under drought stress condition are considered an external indicator of metabolism modifications in the cells of plants. Previously, several studies have reported the damaging effects of drought on the growth parameters and yields of many crop plants^25,39,46–48^. Nevertheless, the range of losses under drought-stressed condition varied with the severity of stress and plant growth phases. Moreover, it has also been reported that SA, Zn and GB applications may play important role in improving the growth characters, yield, and yield-related components under diverse stresses in various plant species ^17,23,27,35,49^.

In the present study, the chlorophyll contents and RWC were significantly affected in maize plants under drought stress. Nevertheless, the foliar application of SA, Zn, and GB treatments improved these parameters under both soil water condition (Fig. 1). Several previous researches have demonstrated that the reduction in chlorophyll contents is one of the important essential factors which could restrict the photosynthesis process under drought stress^38,50–52^. Rahmani et al.^53^ mentioned that the RWC and chlorophyll content were decreased under drought, but the foliar application of Zn statistically improved the drought-tolerance by promotion in the RWC and chlorophyll content under water deficit condition during the seed-filling stage in safflower plants. The decreases in chlorophyll contents under the drought-stressed condition in the current investigation are consistent with Yavas and Unay^54^ who indicated that the drought stress at the grain-filling stage considerably decreased chlorophyll contents and RWC, while the foliar applications of Zn and SA had a positive impact on RWC and chlorophyll content and mitigated the damaging effects of drought stress on plants. The RWC is considered a useful variable to appraise the physiological water status in the leaves of plants^55^_._ Moharramnejad et al.^52^ indicated that drought stress remarkably decreased the contents of Chl. a and total Chl., and RWC when compared to the normal condition. Previously, many studies reported that the exogenous applications of SA, Zn, and GB treatments enhanced the Chl. a, Chl. b and total Chl. contents, and leaf RWC in different crops^35,42,46,56^.

In the current study, the leaf gas-exchange attributes were substantially affected under the drought-stressed condition. All photosynthetic gas-exchange parameters were increased by SA, Zn, and GB foliar applications except for the intercellular CO_2_ concentration (Fig. 2). The net photosynthesis rate, transpiration rate, and stomatal conductance were declined under drought stress, while the spraying applications of GB, Zn, and SA increased CO_2_ assimilation, enhanced physiological water status, and improved the synthesis of metabolites in many crop plants^51,57,58^. Habibi^46^ demonstrated that the net photosynthetic rate, transpiration rate, and stomatal conductance were significantly reduced under drought stress, but the spraying application of SA treatment statistically increased them as compared to control in drought-stressed barley plants. The application of adequate Zn substantially enhanced the leaf CO_2_ net assimilation rate and the efficiency of photosystem II under drought stress in chickpea plants^42^.

In this study, drought stress statistically increased the activity of antioxidant enzymes including APX, GR, POD, CAT, and SOD (Fig. 3). Nevertheless, the increases in the activity of antioxidant enzymes were not sufficient to protect against the accumulation of ROS and were not adequate to reform the injuries of the oxidative stress caused by the drought stress. These antioxidant enzymes were increased by the foliar application of SA, Zn, and GB treatments under the drought-stressed condition in maize plants (Fig. 3). Dianata et al.^59^ revealed that the exogenous treatments of SA significantly enhanced the activities of antioxidant enzymes (SOD, POD, and CAT) under drought stress as compared with no SA treatment. However, Ma et al.^37^ found that the relative gene expression levels of APX, GR, CAT, and APX enzymes were significantly increased, but the content of MDA was substantially decreased by the application of Zn under moderate and severe droughts for 10 and 20 days after initiation of stress conditions in wheat flag leaves. The exogenous applications could amend enzymatic antioxidants in maize plants under different soil water conditions and could efficiently scavenge harmful ROS which was showed by a noticeable decrease in the contents of MDA, H_2_O_2_, and O_2_^−^ in these plants (Figs. 3, 4). Previously, many studies also recorded that enzymatic and non-enzymatic antioxidants were elevated, but the H_2_O_2_, O_2_^−^, and MDA contents were reduced by spraying applications under abiotic stresses in various crops^31,52,60–62^. The SOD enzyme is regarded as the first line in protecting against ROS accumulation, which converts the superoxide radical to oxygen and hydrogen peroxide^63^. Asada^64^ illustrated that the enzyme of CAT induced the conversion of hydrogen peroxide to water and molecular oxygen, where hydrogen peroxide was regarded as a powerful and harmful oxidizing agent. Mittler^45^ indicated that the MDA content was considered an adequate indicator of lipid peroxidation in membranes of cells. In this study, the content of MDA in maize leaves was statistically elevated by drought stress and it was concomitant with increased contents of H_2_O_2_ and O_2_^−^, while exogenous applications of SA, Zn or GB reduced the contents of MDA, H_2_O_2_, and O_2_^−^ (Figs. 3, 4). The present results indicated that the foliar applications gave plants a high ability to deal with oxidative stress and could enhance the drought-tolerance in plants. Consistently, several previous studies have documented that the foliar application of different compounds increased the antioxidant enzymes and decreased the content of MDA in various crops ^17,42,56,65^.

The results indicated that the accumulations of ROS were significantly increased under drought stress but they were reduced by the foliar SA, Zn, and GB treatments (Fig. 4). Our results are consistent with many previous studies. who revealed that the accumulations of ROS were substantially elevated under drought stress, but the foliar applications of SA, Zn, and GB treatments reduced the accumulations of ROS in different crop plants^31,41,62,66–68^.

In addition to the defense system of antioxidant enzymes, osmoregulation is strongly involved in drought amelioration through adjusting the osmotic stress. The compounds of proline and soluble sugar are very important for the osmoregulation process in plants under drought stress. In the current study, proline and soluble sugar in maize leaves were substantially increased under the drought stress and they were statistically increased by foliar application of SA, Zn, and GB (Fig. 5). This phenomenon could be considered as a mechanism that water loss in plants was inhibited by modification of the osmotic condition. These results are in harmony with previous researchers^51,59,68–70^, who found that free proline content and total soluble sugar were increased under drought stress in different crop plants. Upadhyaya et al.^48^ documented that Zn treatment significantly increased the leaf proline content under drought stress when compared with the control plants. Supporting our findings, Osman^43^ pronounced that the foliar application of GB considerably promoted the accumulations of total soluble sugars and free amino acids under drought stress at different growth phases in pea plants. Also, Aldesuquy et al.^71^ mentioned that the exogenous treatment of SA statistically increased soluble sugars and proline content in flag leaf during the reproductive stage under drought stress conditions when compared to untreated wheat plants.

**Figure 6:**
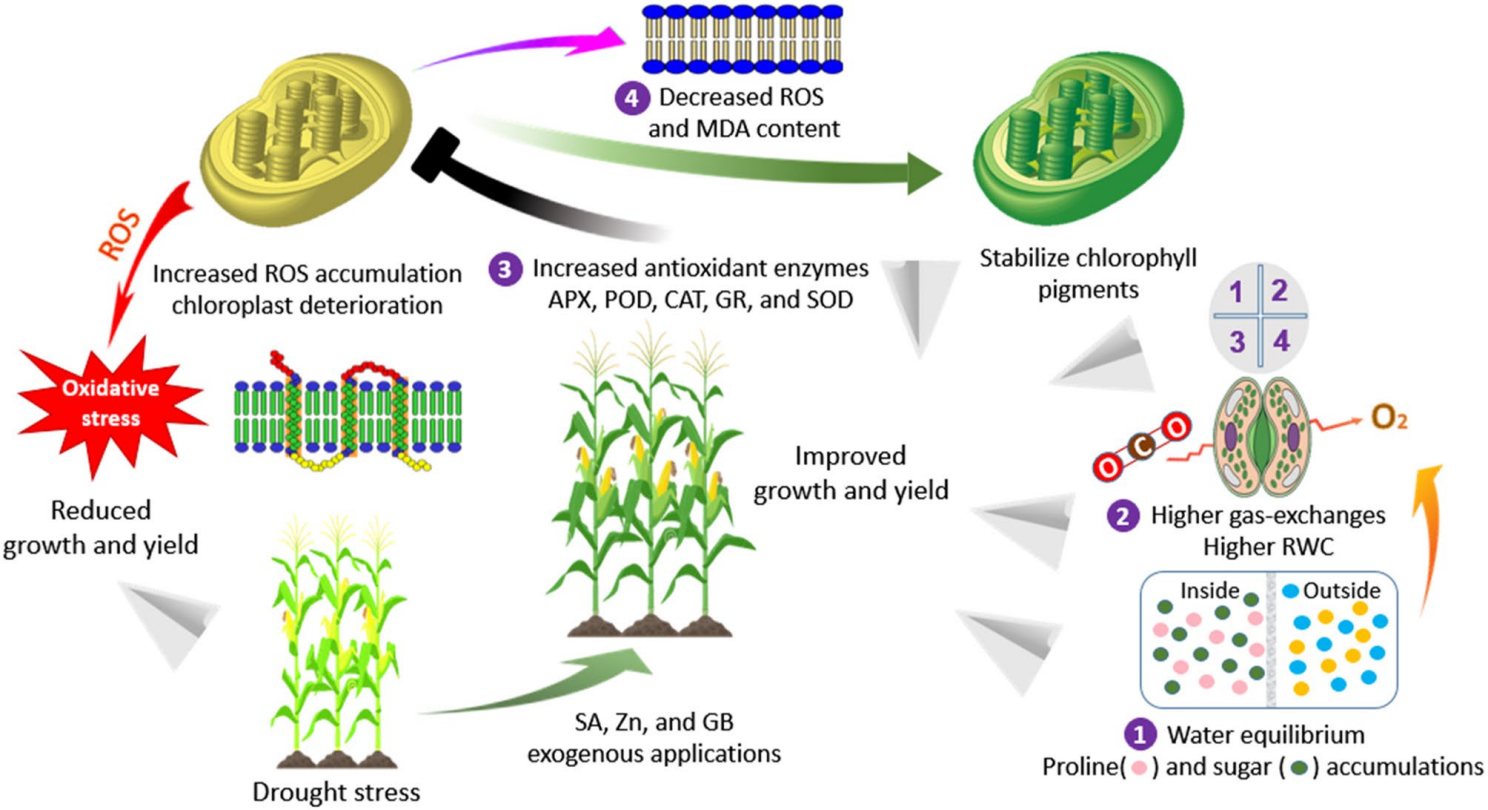
The different mechanisms of exogenous salicylic acid (SA), zinc (Zn), and glycine betaine (GB) applications mediated drought-tolerance in maize crop. ROS, reactive oxygen species; APX, ascorbate peroxidase; GR, glutathione reductase; POD, peroxidase; CAT, catalase; SOD, superoxide dismutase; MDA, malonaldehyde; RWC, relative water content.

Based on all the above results, the beneficial effects of SA, Zn, and GB treatments could be ascribed to decrease the accumulation of H_2_O_2_, O_2_^−^, and MDA contents, perhaps by increasing the activity of antioxidant enzymes and enhancing the osmolytes accumulation (Fig. 6). Moreover, the exogenous applications of SA, Zn, and GB were effective in improving the leaf gas-exchange attributes and RWC, and stabilizing chlorophyll pigments (Fig. 6). These mechanisms are very important to sustain maize production in water deficit conditions.

## Materials and methods

### Experimental design and plant growth conditions

The controlled pot trial was carried out during the summer growing season of 2019 at the glasshouse of the College of Agronomy and Biotechnology (CAB), Southwest University (SWU), Chongqing, China. The experimental area lies at longitude 106^◦^ 26′ 02′' E, latitude 29^◦^ 49′ 32′' N, and altitude 220 m. During the growing season, the average minimum and maximum temperatures were 24 °C and 35 °C, and the relative humidity was between 76 and 84%. The experiment was performed in a completely randomized design (CRD) with two factors: two soil water conditions and four spraying treatments. The experiment comprised eight treatments, and each treatment contained ten pots and three replications. Summer maize cultivar Xinzhongyu 801, a commonly cultivated hybrid in China, was selected for this study. It has high-yield, stability, and adaptability and is widely grown in south-west China. Each plastic pot (30 cm diameter, 35 cm depth) was filled with 15 kg air-dried and sieved (0.5 mm) soils, which was collected from the experimental station at the CAB, SWU. Experimental soil was clay loam, and had the following physical and chemical properties: pH of 6.25, organic matter of 12.58 g kg^−1^, electrical conductivity (EC) of 0.45 ds m^−1^, bulk density of 1.44 g cm^−3^, soil water content at field capacity (FC) of 24.35%, total N of 0.98 g kg^−1^, available phosphorous of 15.53 mg kg^−1^, and available potassium of 86.11 mg kg^−1^. During the time of soil filling, the fertilizers including 5.4 g controlled-release urea (44.6% N), 10 g calcium superphosphate (12% P_2_O_5_), and 2 g potassium chloride (60% K_2_O) were applied to each pot. Five uniform grains were manually cultivated on the 10^th^ of April in all pots at a depth of 4–5 cm. Thinning processes were performed after one week from germination, and two uniform seedlings pot^−1^ were selected for the subsequent studies. Thus, each treatment had 20 plants. All pots were irrigated to 85% FC from the tap water till the start of drought stress treatments.

### Soil water conditions

The plants were subjected to two soil water conditions for 28 days, from the fourteenth leaf (V_14_) until blister (R_2_) growth stages of maize: well-watered condition (85% of field capacity; WW) and drought-stressed condition (50% of field capacity; WD). During the drought period, the pots were weighed every day to keep the required water levels in the soils by adding proper water volumes. Soil water contents for 85% and 50% field capacity were 22.5% and 11.25%, respectively. The weights similar to each of the following soil water contents (22.5% and 11.25%) are 17.45 and 16.30 kg/pot, respectively. These two field capacities are very important and we applied 85% as a normal condition treatment which maintains soil moisture near-maximum water-holding capacity and 50% as a drought condition which is near-minimum water-holding capacity.

Soil water content (SWC) was computed by using the following equation: SWC % = [(FW-DW)/DW] × 100, where FW was the fresh weight of soil sample from the inner area of each pot and DW was the dry weight of soil sample after oven drying at 85 °C for 3 days^72^.

### Spraying treatments

After 7 and 14 days of drought imposition, the maize plants under each soil water condition were sprayed with double distilled water (CK), 140 mg l^−1^ SA (2-hydroxybenzoic acid, C_7_H_6_O_3_, MW = 138.12 g mol^−1^, pH: 5.8), 4 g l^−1^ Zn (zinc sulfate heptahydrate, ZnSO_4_ ‧7H_2_O, MW = 287.54 g mol^−1^), and 11.5 g l^−1^ GB (betaine, C_5_H_11_NO_2_, MW = 117.14 g mol^−1^). SA was added in ethanol to increase the solubility in water (1 g 10 ml^−1^)^73^. Treatments were applied on all plants at the sixteenth leaf (V_16_) and eighteenth leaf (V_18_) growth stages. We finished the spraying of treatments before tasseling stage. Tween-20 (0.05%) was added with spraying treatments as a surfactant at the time of applications. we sprayed two times to ensure absorption spraying treatments into plants leaves after imposition of drought. The effective concentrations of these spraying treatments were selected based on results of previous studies carried out under drought stress^17,33,54,56,74–76^.

### Plant sampling and analyses

Maize plants were sampled after 14 days of spraying applications (28 days of drought imposition) to measure growth parameters, chlorophyll contents, RWC, leaf gas-exchange attributes, and biochemical features. Completely expanded, undamaged, and healthy maize plant leaves (3^rd^ leaf from the top) were sampled from all repetitions. After cleaning, the leaves of maize plants were frozen with liquid N_2_ immediately and stored at -80 °C for biochemical analyses, and the kits were purchased from Sino Best Biological Co., Ltd., China. The yield and its components were recorded at harvest.

### Growth parameters, yield, and its components

Six maize plants were randomly selected to measure plant height, fresh weight plant^−1^, dry weight plant^−1^, number of leaves plant^−1^, and leaf area plant^−1^. Plant height was measured by a meter scale, biomass accumulation was determined by an electronic weighing balance, and total leaf area plant^−1^ was measured by using a LI-3100 laser area meter (Li-COR, CID, Inc., USA). The dry weight plant^−1^ was estimated following oven drying at 85 °C for 48 h. At full maturity (plants at 120-days old), six maize plants were randomly harvested to measure 100-grain weight (g), the number of grains plant^−1^, biological yield (g plant^−1^), grain yield (g plant^−1^), and harvest index (HI). The HI was computed as the percent ratio of grain yield and biological yield according to Donald^77^.

### Chlorophyll contents and RWC

Contents of chlorophyll (Chl. a, Chl. b and total Chl.) in the 3^rd^ leaf from the top were determined according to Peng and Liu^78^. Extraction of a 250 mg leaf blade sample was done with 10 ml ethanol-acetone (vol. 1:2 ratio), and the extract was moved to a 15 ml centrifuge tube. The tubes were put in the dark to avoid from light for 24 h until the sample changed into a white color. The chlorophyll contents were calculated by the following equations: Chlorophyll a content (mg/g tissue) = (12.7D_663_—2.69D_645_) × V/ (1000 × W), Chlorophyll b content (mg/g tissue) = (22.7 D_645_—4.68D_663_) × V/ (1000 × W), and total chlorophyll content (mg/g tissue) = (20.2 D_645_ + 8.02 D_663_) × V/ (1000 × W), where, D_663_ and D_645_ are the corresponding wavelengths of the optical density value (nm), V is the volume of extracting liquid (cm^3^) and W is the weight of fresh leaf (mg). The relative water content (RWC) of maize leaves was measured according to Barrs and Weatherley^79^. Fresh maize leaves were cut into small segments (1.5 cm length), and weighed fresh weight (FW, mg); Later these leaves were floated in distilled water for 4 h under low light to register saturated weight (SW, mg), and then dried in an oven until constant weight at 80 °C for 24 h to record dry weight (DW, mg). RWC was computed as: RWC (%) = (FW—DW)/ (SW—DW) × 100%.

### Leaf gas-exchange attributes

Photosynthesis characteristics including the net photosynthesis rate (Pn), stomatal conductance (Gs), transpiration rate (Tr), and intercellular CO_2_ concentration (Ci) were recorded using a portable infrared gas analyser based photosynthesis system (LI-6400; LiCor, Inc., Lincoln, NE, USA) at 09:30–11:30 am from the fully expanded leaf (3^rd^ leaf from top). Air relative humidity and ambient CO_2_ concentration were about 78% and 370 µmol CO_2_ mol^−1^, respectively during collecting the data.

### Assay of antioxidant enzymes activity and lipid peroxidation

The activities of different enzymatic antioxidants in maize leaves were recorded using commercial kits as per the manufacturer’s instructions. The kits for superoxide dismutase (SOD-A500), catalase (CAT-A501), ascorbate peroxidase (APX-A304), glutathione reductase (GR-A111), and peroxidase (POD-A502) were purchased from the same company as indicated above. The absorbance readings of SOD, CAT, APX, GR, and POD were detected at 560 nm, 240 nm, 290 nm, 340 nm, and 470 nm, respectively using an ultraviolet (UV)-visible spectrophotometer, and the activities of these enzymes were expressed as units per fresh weight (U g^−1^ FW). The units of the antioxidant enzymes activity were defined as follows: “one unit of GR activity was expressed as the amount of enzyme depleting 1 µmol NADPH in 1 min, one unit of SOD activity was defined as the amount of enzyme needed to reduce the reference rate to 50% of maximum inhibition, one unit of CAT activity was measured as the amount of enzyme that decomposes 1 nmol H_2_O_2_ at 240 nm min^−1^ in 1 g fresh weight, one unit of APX was estimated as the amount of enzyme required for catalyzing 1 μmol ASA at 290 nm 2 min^−1^ of 1 g fresh weight in 1 ml of a reaction mixture, and one unit of POD activity was demonstrated as the absorbance change of 0.01 at 470 nm min^−1^ for 1 g fresh weight in 1 ml of a reaction mixture”^80–82^. Lipid peroxidation was assayed as MDA content in maize leaves, through thiobarbituric (TBA) method using MDA Detection Kit (A401), obtained from the same company as indicated above. The absorbance for MDA was recorded at 532 and 600 nm and expressed as nmol g^−1^ fresh weight.

### Estimation of reactive oxygen species accumulation

The contents of hydrogen peroxide (H_2_O_2_) and superoxide anion radical (O_2_^−^) in the leaves of maize were recorded using the commercial ‘H_2_O_2_ Detection Kit (A400)’, and ‘O_2_^−^ Detection kit (A407)’, respectively, being purchased from the same company as indicated above. H_2_O_2_ content was estimated at 415 nm and represented as μmol g^−1^ fresh weight. Super oxygen anion serotonin reacted with hydrochloride to produce NO_2_^−^. The NO_2_^−^ entered with the interaction of amino benzene and alpha-pyridoxine to the production of red compounds at 530 nm which had a characteristic absorption peak. The content of O_2_^−^ was measured at 530 nm, and expressed as μmol g^−1^ fresh weight.

### Determination of osmolytes accumulation

The contents of proline and soluble sugar in maize leaves were determined using commercial kits according to the manufacturer’s instructions. The kits for proline (PRO, A605), and soluble sugar contents (SSC, B602) were purchased from the same company as indicated above. The absorbance readings of the toluene layer were read on a spectrophotometer at 520 nm. Proline (Sigma, St Louis, MO, USA) was used as a standard curve. Proline content was expressed as µg g^−1^ fresh weight. The absorbance readings of SSC was detected at 620 nm using an ultraviolet (UV) visible spectrophotometer. Soluble sugar content was articulated as mg g^−1^ fresh weight.

### Statistical analysis

Data were statistically analyzed following the analysis of variance (ANOVA) technique according to the Two-way factorial Design using Statistical Software Package MSTAT-C^83^. Least significant differences test (L.S.D) at 5% probability was used to test the significance among mean values of each treatment^84^. Ten pots and three replications were used for each treatment and 20 plants were grown for each treatment. Sigma Plot 10.0 (Systat Software Inc., San Jose, CA, USA) was used for graphical presentation of the data.

## Conclusion

This experiment revealed that the spraying application of 140 mg l^−1^ SA, 4 g l^−1^ Zn, and 11.5 g l^−1^ GB improved drought-tolerance in maize crop through increased photosynthesis pigments and RWC, improved leaf gas-exchange attributes, enhanced activities of antioxidant enzymes, reduced of MDA, H_2_O_2_, and O_2_^−^ contents, and higher accumulation of osmolytes in drought-stressed plants. Thus, it might be considered as an important strategy to improve the plant growth parameters, yield, and its components under drought-stressed condition. Overall, GB application was the most effective followed by SA and Zn applications to alleviate the injurious effects in maize triggered by drought.
